# Monitoring Dynamic Changes of Cellular Membrane GSH During Stroke via an ESIPT-Based Near-Infrared Fluorescent Probe

**DOI:** 10.3390/molecules30030592

**Published:** 2025-01-28

**Authors:** Yue Gao, Zhao Wang

**Affiliations:** Hubei Provincial Engineering Research Center of Racing Horse Detection and Application Transformation, Equine Science Research and Horse Doping Control Laboratory, College of Food Science and Technology, Wuhan Business University, Wuhan 430056, China; gaoyue@m.scnu.edu.cn

**Keywords:** near-infrared probe, excited-state intramolecular proton transfer, glutathione, ferroptosis, stroke

## Abstract

Stroke, primarily ischemic (85%), results from inadequate blood supply and is worsened by ferroptosis, characterized by free radical generation and lipid peroxidation. Monitoring ferroptosis is essential for understanding its mechanisms and developing treatments. Glutathione (GSH) is a key ferroptosis biomarker, but current probes are limited by short excitation/emission wavelengths, small Stokes shifts, and inability to monitor dynamic GSH changes at the cellular membrane, where ferroptosis plays a crucial role. To address these issues, we developed the PM-Red-GSH, a novel near-infrared (NIR) probe based on the Excited-state intramolecular proton transfer (ESIPT) mechanism. It shows strong NIR emission (715 nm), large Stokes shift (290 nm), and enhanced membrane binding (PCC = 0.95) due to its alkyl group. PM-Red-GSH enables dynamic GSH monitoring in an MCAO mouse model. These findings offer new insights into ferroptosis and stroke treatment.

## 1. Introduction

Stroke is a neurological disorder caused by inadequate blood supply, leading to rapid depletion of oxygen and glucose in brain tissue, thereby triggering a cascade of neurological dysfunctions [1]. Based on its pathophysiological mechanisms, stroke can be categorized into ischemic and hemorrhagic types, with ischemic stroke accounting for approximately 85% of all cases [2]. Ischemic stroke typically results from cerebral vessel obstruction, leading to the cessation of blood flow to local brain regions [3]. This interruption in blood supply induces energy metabolic disturbances, which rapidly impair neuronal function. Prolonged ischemia may lead to irreversible cell necrosis. Ferroptosis, a distinct form of regulated cell death, plays a critical role in exacerbating stroke-induced injury [4,5]. This process is driven by iron metabolism dysregulation, free radical generation, and lipid peroxidation, which collectively damage cellular structures and amplify stroke pathology [6,7,8]. Therefore, monitoring ferroptosis during stroke is essential for understanding its underlying mechanisms and for the development of targeted therapeutic strategies.

Recent studies have identified glutathione (GSH) as a crucial biomarker for monitoring ferroptosis, as it regulates the production of phospholipid peroxides through the System Xc^−^/GSH/GPX4 system [9,10]. Fluorescence-based imaging techniques, known for their high sensitivity, selectivity, ease of use, real-time capabilities, high resolution, and non-invasiveness, have emerged as powerful tools for tracking GSH fluctuations in complex biological systems [11,12,13,14,15]. Although previous research has employed GSH probes to analyze ferroptosis during stroke, significant limitations remain: (1) Short excitation and emission wavelengths hinder detection depth and sensitivity; (2) Small Stokes shifts (<100 nm) reduce signal clarity and resolution; and (3) Traditional GSH probes primarily monitor intracellular GSH concentrations, without effectively detecting dynamic GSH changes at the cellular membrane. As the cellular membrane is a key site of ferroptosis, the ability to track membrane-bound GSH changes is crucial for understanding the mechanism of this process [16,17,18,19,20,21,22,23,24,25,26,27,28,29,30,31,32,33].

To address these challenges, we have developed a novel near-infrared (NIR) fluorescent probe, PM-Red-GSH, based on the Excited-State Intramolecular Proton Transfer (ESIPT) mechanism. This probe exhibits excellent NIR emission at 715 nm and a substantial Stokes shift of 290 nm. A 2,4-dinitrobenzene sulfonyl group serves as the GSH recognition unit, while a silane moiety enhances its lipophilicity, thereby facilitating stronger membrane association (PCC = 0.95). PM-Red-GSH enables sustained imaging of cellular membrane dynamics during ferroptosis. Furthermore, it enables the dynamic monitoring of GSH levels in the brain of a middle cerebral artery occlusion (MCAO) mouse model. These findings provide novel insights into the study of ferroptosis mechanisms and suggest new directions for the development of therapeutic strategies for stroke.

## 2. Results and Discussion

### 2.1. Design Strategy and Synthesis Process of Fluorescent Probe PM-Red-GSH

**Scheme 1:**
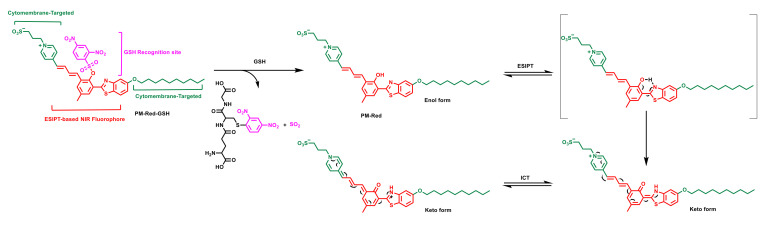
The sensing mechanism of PM-Red-GSH with GSH.

In this study, we designed and synthesized a novel near-infrared fluorescent probe, PM-Red-GSH. As illustrated in Scheme 1, the probe integrates a large π-conjugated system functioning as a near-infrared fluorophore, two cytomembrane-targeting moieties, and 2,4-dinitrobenzenesulfonyl ether, which serves as both a fluorescent quencher and a GSH recognition trigger. This molecular architecture facilitates the near-infrared fluorophore’s ability to manifest the ESIPT effect (Scheme 1), thereby shifting the emission to longer wavelengths (715 nm) and yielding a substantial Stokes shift (290 nm), along with an exceptional quantum yield (Φ = 11.4%, where Φ denotes the quantum yield). These characteristics effectively minimize crosstalk between excitation and emission spectra, as well as self-absorption, enhancing its suitability for in vivo imaging in murine models.

The cytomembrane-targeting moieties are composed of propionine sulfonate and decyl ether. The propionine sulfonate pyridine salt not only facilitates cytomembrane targeting but also enhances the water solubility of the probe. The decyl ether, a hydrophobic group, further augments the probe’s affinity for cell membranes. The 2,4-dinitrobenzenesulfonyl ether functions as the GSH recognition site, where electrostatic interactions enable selective detection and recognition of GSH.

Thus, we have developed PM-Red-GSH, a novel near-infrared fluorescent probe activated by GSH. This probe effectively targets the cytomembrane and enables real-time monitoring of GSH fluctuations during ferroptosis in live cells and in a middle cerebral artery occlusion (MCAO) model. The detailed synthesis procedures, along with the ^1^H NMR, ^13^C{^1^H} NMR, and HR-MS spectra for each compound, are provided in Figures S1–S15.

### 2.2. Spectral Response Performances of PM-Red-GSH Toward GSH

**Figure 1:**
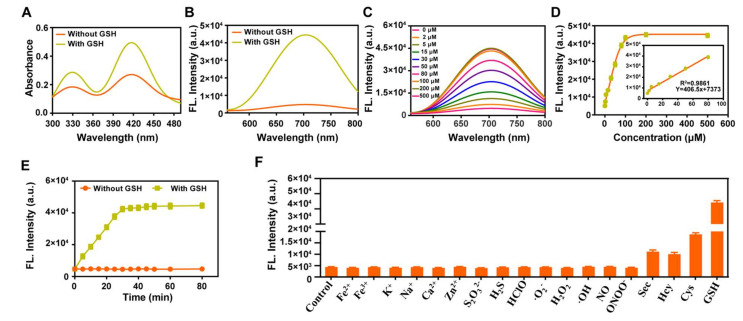
Absorption spectra (**A**) and Fluorescence spectra (**B**) of PM-Red-GSH in solution, with (Yellow line) and without (Red line) GSH. (**C**) Fluorescence spectra of PM-Red-GSH at varying GSH concentrations (0–500 µM), excited at λ = 425 nm. (**D**) Fluorescence intensity at 715 nm as a function of GSH concentration. Inset: Linear correlation between fluorescence intensity at 715 nm and GSH concentration in the range of 0–80 µM. (**E**) Time-dependent fluorescence intensity of PM-Red-GSH incubated with 0 µM and 100 µM GSH. (**F**) Fluorescence response of PM-Red-GSH (10 µM) upon incubation with 500 µM GSH and various other analytes (200 µM).

The spectral properties of PM-Red-GSH were systematically examined in a PBS-CH_3_CN solution (pH = 7.4, containing 50% CH_3_CN). The probe exhibited two absorption bands centered at 330 nm and 425 nm. Upon reaction with GSH, the probe maintained these two absorption peaks at the same wavelengths (Figure 1A and Table S1 in the Supplementary Materials). Under excitation at 425 nm, PM-Red-GSH displayed weak fluorescence at 715 nm. However, upon interaction with 100 µM GSH, the fluorescence intensity at 715 nm increased significantly (Figure 1B).

The absorption spectroscopy experiments revealed that, within the concentration range of 1, 2, 3, 4, 6, 8, 10, 15, and 20 µM, the absorption intensity exhibited a linear relationship, indicating that PM-Red-GSH maintains excellent solubility within this concentration range (Figure S16). To assess the sensitivity of PM-Red-GSH to GSH, a series of experiments were conducted in which 10 µM of PM-Red-GSH was incubated with varying concentrations of GSH (0–500 µM) in PBS buffer at 37 °C. As the GSH concentration increased, the fluorescence intensity at 715 nm gradually intensified. When 100–500 µM GSH was added, the fluorescence intensity reached a plateau, with the maximum fluorescence enhancement increasing by 15-fold (Figure 1C,D). The probe exhibited a linear fluorescence response to GSH concentrations ranging from 0.15 to 80 µM, with a correlation coefficient (R^2^) of 0.986 and a slope of 406.5 (Figure 1D, inset). This enabled the calculation of the limit of detection (LOD) for PM-Red-GSH towards GSH, which was determined to be 41 nM, using the formula LOD = 3σ/k, where σ represents the standard deviation of 10 blank samples and k denotes the slope of the linear regression. Additionally, PM-Red-GSH was shown to respond to GSH within 30 min (Figure 1E).

To evaluate the selectivity of the probe for GSH, comprehensive studies were performed with other analytes, including Sec, Hcy, Cys, GSH, metal ions (Fe^2+^, Fe^3+^, K^+^, Na^+^, Ca^2+^, Zn^2+^), sulfides (S_2_O_3_^2−^, H_2_S), and various reactive oxygen and nitrogen species (ROS/RNS), such as HClO, ·O_2_^−^, H_2_O_2_, ·OH, NO, and ONOO^−^ (Figure 1F). Fluorescence spectra were recorded after the addition of these substances to the probe solution. Notably, only GSH induced a significant fluorescence enhancement, while other analytes caused negligible changes in the fluorescence signal. These findings unequivocally confirm that PM-Red-GSH exhibits a high degree of selectivity for GSH detection.

Finally, the probe’s pH sensitivity was investigated. At pH values ranging from 5.0 to 8.5, the fluorescence of the probe remained largely unchanged. Moreover, the fluorescence increase upon GSH addition in this pH range was nearly identical, indicating that the probe’s response to GSH is pH-independent within the physiological pH range of 5.0–8.5 (Figure S17). These results confirm that PM-Red-GSH specifically responds to GSH with high selectivity, effectively distinguishing it from other biological ions, reducing agents, ROS, and RNS under physiological conditions.

### 2.3. Bioimaging Application of PM-Red-GSH in Living Cells

After demonstrating the favorable response of the probe PM-Red-GSH to GSH, we further explored its potential for in vivo imaging of GSH by conducting cytotoxicity assays. As shown in Figure S18, following co-incubation with various concentrations of PM-Red-GSH (0–30 µM) for 24 h, MTT analysis revealed that the survival rate of BV-2 cells remained above 85%. These results suggest that PM-Red-GSH exhibits low cytotoxicity toward BV-2 cells, making it suitable for intracellular GSH detection.

Next, we employed the probe to assess GSH levels during the ferroptosis process. Initially, we examined the imaging capability of the fluorophore PM-Red in BV-2 cells. The red fluorescence signal from the probe strongly overlapped with the green fluorescence signal of DiO (Cell Membrane Green Fluorescent Probe) [34,35,36], yielding a Pearson correlation coefficient of 0.95. This indicates that the fluorophore effectively targets the cell membrane (Figure S19).

Further investigation of GSH detection in living cells was carried out. As depicted in Figure S20, weak red fluorescence was observed in BV-2 cells after 40 min of incubation with PM-Red-GSH, attributed to the endogenous GSH present within the cells. When cells were pretreated with 0.5 mM N-ethylmaleimide (NEM, a thiol scavenging agent) [37] for 40 min prior to incubation with PM-Red-GSH, minimal fluorescence was detected in the red channel. To validate the feasibility of detecting exogenous GSH, BV-2 cells were incubated with PM-Red-GSH (10 µM) for 10 min, followed by treatment with NEM (0.5 mM) and varying concentrations of GSH (0, 5, 10, 20 µM) for 40 min. At a GSH concentration of 20 µM, a 5-fold increase in cellular fluorescence was observed, confirming the probe’s ability to detect exogenous GSH (Figure S20).

**Figure 2:**
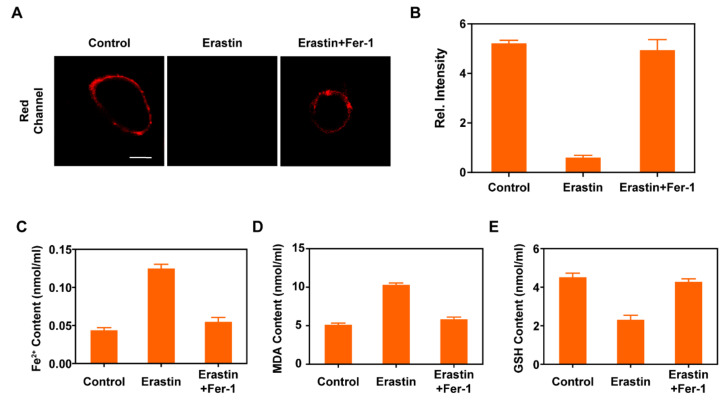
Confocal images of GSH in ferroptosis. (**A**) BV-2 cells under different conditions: control group (only PM-Red-GSH, 10µM); PM-Red-GSH (10 µM) + Erastin (10 µM); PM-Red-GSH (10 µM) + Erastin (10 µM) + Fer-1 (5 mM). (**B**) Histograms of average fluorescent intensities in (**A**) difference was analyzed. Scale bars:20 µm. (**C**) the content of Fe^2+^; (**D**) the content of MDA; (**E**) the content of GSH.

Additionally, a ferroptosis model was established by incubating BV-2 cells with the ferroptosis inducer Erastin. As shown in Figure 2, the fluorescence intensity in the red channel was significantly reduced compared to the control group (only PM-Red-GSH at 10 µM), suggesting that endogenous GSH was consumed during the ferroptosis process. However, the addition of the ferroptosis inhibitor ferrostatin-1 (Fer-1) [38] resulted in a marked increase in red channel fluorescence intensity, indicating that PM-Red-GSH is capable of in situ imaging of ferroptosis.

**Figure 3:**
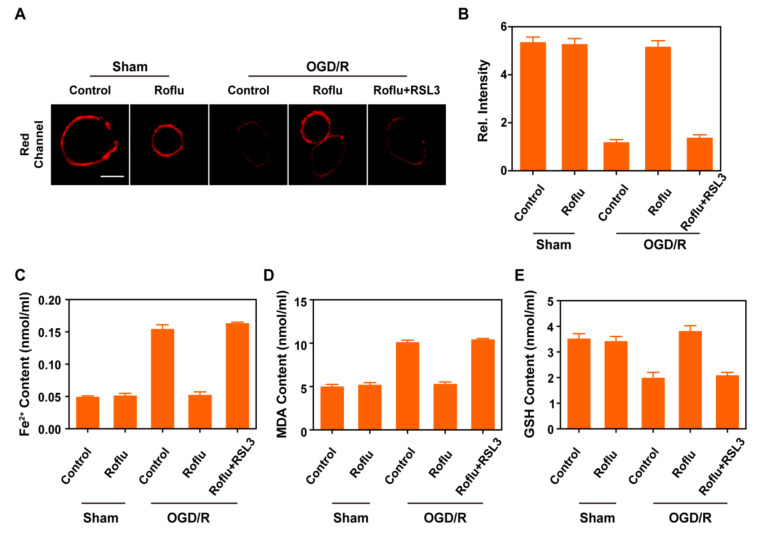
(**A**) Representative confocal images of BV-2 cells subjected to the oxygen-glucose deprivation/reoxygenation (OGD/R) model. The sham group was divided into two conditions: control (untreated BV-2 cells) and Roflumilast (Roflu) treatment (BV-2 cells treated with 10 µM Roflu). The OGD/R group consisted of three conditions: control (OGD-treated cells), Roflu (OGD-treated cells with 10 µM Roflu), and Roflu + RSL3 (OGD-treated cells with 10 µM Roflu and 1 µM RSL3). (**B**) Quantification of the average fluorescence intensity from the confocal images shown in panel (**A**). (**C**) Quantification of Fe^2+^ levels in each experimental group. (**D**) Measurement of malondialdehyde (MDA) content. (**E**) Quantification of glutathione (GSH) levels. Scale bar = 20 µm.

Furthermore, PM-Red-GSH was further employed to detect GSH levels during the oxygen-glucose deprivation/reoxygenation (OGD/R) process [39,40,41], a condition closely linked to ferroptosis. BV-2 cells were subjected to OGD/R to simulate ischemia-reperfusion, and the ferroptosis process was monitored using PM-Red-GSH. As illustrated in Figure 3A,B, the sham group was subdivided into two experimental conditions. The first group, comprising BV-2 cells, served as the control group, with no interventions, while the second group consisted of BV-2 cells treated with Roflumilast (Roflu, a specific drug for stroke). In the sham group, strong red fluorescence was observed, indicating that Roflu treatment did not interfere with the cellular integrity of BV-2 cells. When PM-Red-GSH was used to monitor ferroptosis during OGD/R, a notable reduction in fluorescence intensity in the red channel was observed in the OGD/R groups (control and Roflu + RSL3), in comparison to the sham group. To confirm that the observed fluorescence decrease was attributed to a reduction in GSH levels, cells were pre-treated with 10 µM Roflu. As anticipated, the Roflu-treated cells exhibited sustained strong fluorescence, while the fluorescence in the control and Roflu + RSL3 groups was significantly diminished. These findings unambiguously demonstrate that the PM-Red-GSH probe can sensitively detect GSH depletion during the ferroptosis process in the OGD/R model. Moreover, the trends in Fe^2+^, MDA, and GSH levels showed a strong correlation with the fluorescence intensity changes (Figure 3C–E), further validating the probe’s capacity to monitor ferroptosis progression induced by OGD/R.

### 2.4. In Vivo Imaging of Cerebral Apoplexy in Mice

After elucidating the relationship between ischemia-reperfusion and iron deposition, we employed the middle cerebral artery occlusion (MCAO) model to simulate stroke in mice [42]. Prior to imaging, we assessed the biocompatibility of the PM-Red-GSH probe (administered at 200 µM concentration in 100 µL via tail vein injection) in various tissues, including the brain, heart, liver, spleen, lung, and kidneys, by hematoxylin and eosin (H&E) staining. As shown in Figure S21, no significant organ or tissue damage was observed following administration of the PM-Red-GSH probe, indicating that it possesses excellent tissue biocompatibility and is suitable for in vivo imaging analysis in mice.

**Figure 4:**
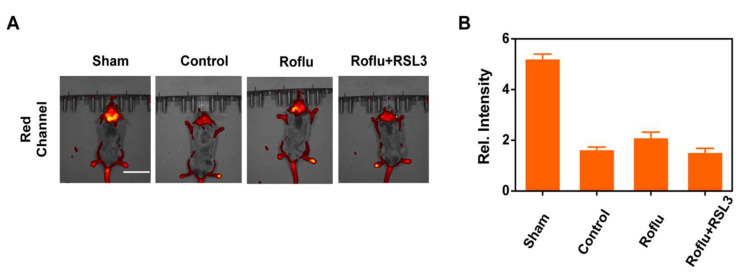
Visual imaging MCAO in living mice model. (**A**) Mice using MCAO model: the sham group (the mice without anything treatment); the control group (the mice undergoing MCAO); the Roflu group (the mice undergoing MCAO and treatment with Roflu); the Roflu+RSL3 group (the mice undergoing MCAO and treatment with Roflu, Roflu + RSL3). (**B**) The average fluorescence intensity of (**A**). Scale bar = 2 cm.

Subsequently, we established the MCAO mouse model to monitor GSH levels in the brain. As depicted in Figure 4A,B, the sham-operated group (normal mice) exhibited strong fluorescence signals in the brain. In contrast, the MCAO control group showed a marked reduction in fluorescence intensity in the brain compared to the sham group. Furthermore, the Roflu+RSL3 group, which received treatment with both Roflu and RSL3, demonstrated a significant decrease in fluorescence intensity compared to the sham group. Notably, the Roflu group, which was treated with Roflumilast alone, exhibited an increase in fluorescence intensity relative to the control and Roflu+RSL3 groups, confirming that the observed fluorescence changes were indeed induced by alterations in GSH levels during the MCAO process.

Taken together, these in vivo results clearly demonstrate that changes in GSH levelsare closely linked to ferroptosis during ischemia-reperfusion. Moreover, the PM-Red-GSH probe proves to be an excellent fluorescence probe for near-infrared imaging of GSH in the MCAO model, offering significant potential for advancing research on stroke and related diseases.

## 3. Materials and Methods

Details on the experimental procedures are provided in the Supplementary Materials.

## 4. Conclusions

In this study, we designed and synthesized a novel near-infrared (NIR) fluorescent probe, PM-Red-GSH, specifically developed for the monitoring of glutathione (GSH) in a middle cerebral artery occlusion (MCAO) mouse model. This probe is based on the electronic transition-induced excited-state intramolecular proton transfer (ESIPT) mechanism, exhibiting exceptional NIR emission at 715 nm and a substantial Stokes shift of 302 nm. The probe incorporates a 2,4-dinitrobenzenesulfonyl group as the GSH recognition unit, while an alkyl group enhances its lipophilicity, promoting strong membrane binding (PCC = 0.95). In vitro experiments demonstrated that PM-Red-GSH exhibits excellent responsiveness to GSH, remarkable pH stability, and low cytotoxicity. Additionally, the probe enables extended (up to 4 h) cellular membrane dynamic imaging during RSL3-induced ferroptosis, providing deep and sustained monitoring of ferroptosis progression. Furthermore, PM-Red-GSH is capable of sensitively detecting GSH depletion during the oxygen-glucose deprivation/reperfusion (OGD/R) model, offering new insights into the mechanisms of ferroptosis. In the MCAO mouse model, PM-Red-GSH successfully monitored GSH level changes in the brain, highlighting its potential as a tool for ferroptosis analysis in stroke models. Moreover, the study demonstrated that Roflumilast (Roflu) significantly protects MCAO mice, alleviating pathological changes induced by stroke, further validating the utility of PM-Red-GSH in the context of stroke and related diseases. In conclusion, the PM-Red-GSH probe developed in this work provides a novel and reliable tool for real-time dynamic monitoring of ferroptosis, with broad applications in the study of stroke, neurodegenerative diseases, and other biomedical fields. This research represents a significant contribution to the advancement of fluorescent probes for bioimaging and holds promising potential for further applications in clinical disease research.

## Data Availability

The datasets collected and/or analyzed during the current study are available from the corresponding author upon reasonable request in compliance with ethical standards.

## Supplementary Materials

The following supporting information can be downloaded at: https://www.mdpi.com/article/10.3390/molecules30030592/s1, Figure S1–S14: NMR spectrum of compounds; Figure S15: High-resolution mass spectrum of PM-Red-GSH; Figure S16: The UV-vis absorption of PM-Red-GSH; Figure S17: pH effects of the probe and the reaction between PM-Red-GSH and GSH; Figure S18: Cytotoxicity of PM-Red-GSH. BV-2 cell; Figure S19: Confocal images of BV-2 cells after co-staining with 10 µM PM-Red and 10µM Dio; Figure S20: (A) Confocal images of BV-2 cells incubated with probe PM-Red-GSH (10 µM) in control group (only probe), PM-Red-GSH (10 µM) for 10 min and then, NEM (0.5 mM), GSH (0, 5, 10, 20 µM) for 40 min. (B) Histograms of average fluorescence intensity of (A). Scale bar = 20 µm; Figure S21: H&E-stained sections of major organs s collected from the control group and probe PM-Red-GSH (100 μL, 200 μΜ) treated group. Scale bar: 50 mm; Table S1: Photophysical properties of PM-RED-GSH under different conditions; Table S2: Comparison of the present probe with other reported GSH fluorescence probe.
