# Supplementary material for: Monitoring Dynamic Changes of Cellular Membrane GSH During Stroke via an ESIPT-Based Near-Infrared Fluorescent Probe
Source: Molecules. 2025 Jan 28;30(3):592. doi: 10.3390/molecules30030592 (PMC11820386; doi:10.3390/molecules30030592)
Supplement: Supplementary file 1 [file molecules-30-00592-s001.zip › molecules-3405374-supplementary.pdf]

molecules

MDPI

Supplementary Materials

Monitoring Dynamic Changes of Cellular Membrane GSH during Stroke via an ESIPT-Based Near-Infrared Fluorescent Probe

Yue Gao [1] and Zhao Wang [1,*]

Hubei Provincial Engineering Research Center of Racing Horse Detection and Application Transformation, Equine Science Research and Horse Doping Control Laboratory, College of Food Science and Technology, Wuhan Business University, Wuhan 430056, China; gaoyue@m.scnu.edu.cn

* Correspondence: wangzhao517@stu.hubu.edu.cn

1. General Information on Materials and Methods

1.1. Instruments and Materials

Unless otherwise stated, all reagents used for the synthesis of the probe were purchased from Aladdin and are of analytical grade. All solvents and reagents were sourced from commercial suppliers and were used without further purification. The reactions were performed in standard glassware. All aqueous solutions were prepared in ultrapure water with a resistivity of 18.25 MΩ·cm (purified by Milli-Q system, Millipore). Column chromatography was performed using silica gel 60 (230 ± 400 mesh, 0.040 ±0.063 mm) from Dynamic Adsorbents. NMR spectra were recorded on a Bruker-500 spectrometer, using TMS as an internal standard. High-resolution mass spectrometry was performed with LTQ FT Ultra (Thermo Fisher Scientific, America) in MALDI-DHB mode. Absorption spectra were recorded with a UV-vis spectrophotometer (Shimadzu UV-2550, Kyoto, Japan), and fluorescence spectra were obtained with a fluorimeter (Shimadzu RF-6000, Kyoto, Japan). Fluorescence imaging of mice was performed on an IVIS Lumina LT Series III small animal optical *in vivo* imaging system (USA) with an excitation filter of 420 nm and an emission filter of 714 nm. Experimental mice were anesthetized on an R500IE anesthesia machine. Living Image 4.5 software (PerkinElmer, Waltham, MA, USA) was used for data analysis.

1.2. Spectroscopic Measurements

A small amount of probe **PM-Red-GSH** was dissolved in DMSO to prepare the stock solutions (5.0×10^{-2} M). Unless otherwise mentioned, all the measurements of probe **PM-Red-GSH** reaction with target tested in PBS buffer (10 mM, pH 7.4, containing 50% CH_3CN). When the probe **PM-Red-GSH** reaction with GSH at 37 °C for 40 min in a thermostat, a 500 μL aliquot of the reaction solution was transferred to a quartz cell with an optical length of 1 cm for the measurement of absorbance or fluorescence. The excitation wavelength was set to 425 nm, with slit widths of 5 nm for both excitation and emission. And testing the selectivity assay was conducted with 200 μM Sec, Hcy, Cys, 200 μM metal ions (Fe^{2+}, Fe^{3+}, K^+, Na^+, Ca^{2+}, Zn^{2+}), sulfide ($S_2O_3^{2-}$, H_2S), 200 μM ROS/RNS (HClO, $\cdot O_2^-$, H_2O_2, ·OH, NO, $ONOO^-$), respectively.

1.3. Quantum Yield Measurements

The measurement of fluorescence quantum yield was measured by using an ethanol solution of rhodamine B as a standard (10 μM, $\Phi_r = 0.71$) and using the following equation.

 https://doi.org/10.3390/molecules30030592

$$\Phi_s = (A_r \cdot F_s \cdot n_s^2)/(A_s \cdot F_r \cdot n_r^2) \cdot \Phi_r \ (A \leq 0.05)$$

where s and r represent the sample to be tested and the reference dye, respectively. A represents the absorbance at the maximum absorption wavelength, F represents the fluorescence spectrum integral at the maximum absorption wavelength excitation, and n represents the refractive index of the sample to be tested or the reference dye solvent.

1.4. Cytotoxicity Assay

The cytotoxicity was evaluated by MTT assay. Briefly, BV-2 cells (from Procell Life Science & Technology Co., Ltd.) were cultured in DMEM in 96-well microplates in an incubator for 24 h. The medium was next replaced by fresh DMEM containing various concentrations of **PM-Red-GSH** (0–30 μM). Each concentration was tested in five replicates. Cells were rinsed twice with phosphate buffer saline (PBS) 24 h later and incubated with 0.5 mg/mL MTT reagent for 4 h at 37 °C. The absorbance at 490 nm was measured by the microplate reader (Synergy 2, BioTek Instruments Inc.). Cell survival rate was calculated by A/A0 × 100 % (A and A0 are the absorbance of the **PM-Red-GSH** and the control group, respectively).

1.5. Cell Culture and Imaging

BV-2 cells were cultured with DMEM supplemented with 10% (*v*/*v*) fetal bovine serum (Gibco), 100 U/mL penicillin, and 100 μg/mL streptomycin in a humidified atmosphere with 5/95 (*v*/*v*) of CO_2/air at 37 °C. One day before imaging, cells were detached with a treatment of 0.2% (*w*/*v*) trypsin-EDTA solution (Gibco) and suspended in culture media. The cell suspension was then transferred to confocal dishes to grow with adherence. For imaging, BV-2 cells at 80% confluence were harvested by scraping and transferred to confocal dishes to grow with adherence.

1.6. Ferroptosis Model

Cells were treated with erastin (10 μM) for appropriate time to induce ferroptosis. After that the culture media were removed, and the cells were washed with serum-free media and then incubated with **PM-Red-GSH** (10 μM) for different treatments. Imaging was performed with the confocal microscope.

1.7. Measurement of the Biomarkers of Ferroptosis

Cellular Fe^{2+} level was measured by using an iron assay kit (Sigma-Aldrich) according to the manufacturer's instructions. Malondialdehyde (MDA, Sigma-Aldrich) level was measured by using an MDA assay kit (Sigma-Aldrich) according to the manufacturer's instructions.

1.8. Calculation of Mean Fluorescence Intensity

The mean fluorescence density was measured by Image-Pro Plus (v. 6.0) and calculated via the equation (mean density = IOD sum/areasum), where IOD and area were integral optical density and area of the fluorescent region.

1.9. OGD/R Model

OGD/R model of cells was performed by oxygen and glucose deprivation/reperfusion. BV2 cells at 80% confluence were harvested by scraping and transferred to confocal dishes to grow with adherence. When the cells are adherent, the culture medium is changed to sugar-free DMEM and cultured in a three-gas incubator for 5 h without oxygen. Afterwards, these cells were incubated with high-glucose DMEM in a 5 % CO_2 and

95% O_2 atmosphere for 5 h. Then, the cells were incubated with **PM-Red-GSH** (10 μM) for 30 min. Wash cells three times with PBS for confocal imaging.

1.10. Middle Cerebral Artery Occlusion (MCAO) Model

MCAO was induced using a previously described method with slight modifications. In brief, C57BL/6J wild-type mice were anesthetized with 5% isoflurane in O_2 by facemask, followed by ligation of the left middle cerebral artery with 6-0 monofilament (Doccol Corp., Redlands, CA, USA). After 1 h of occlusion, the monofilament was removed to initiate reperfusion. A homeothermic heating pad was employed to monitor and stabilize the mice body temperature at 37 ± 0.5 °C. The same procedure, but without monofilament ligation, was performed on sham-operated mice.

1.11. Histological Staining of the Tissue Slices

After imaging, the mice were killed, and the brains and other tissues (heart, liver, spleen, lung, and kidney) were collected for tissue analysis. Through a series of standard procedures, including fixation in 10% neutral buffered formalin, embedding into paraffin and sectioning at 3 μm thickness, the tissues were stained with hematoxylin-eosin (H&E). Thereafter, the prepared slices were examined by a digital microscope.

1.12. In vivo Imaging Studies

All animal procedures were performed in accordance with the Guidelines for Care and Use of Laboratory Animals of South-Central University of Nationalities and experiments were approved by the Animal Ethics Committee of the College of Biology (South-Central University of Nationalities). Wild-type C57BL/6J mice (n = 300; 25–30 g) were purchased from Hubei Experimental Animal Research Center. (Hubei, China; No. 43004700018817, 43004700020932). All animal experimental protocols were approved by the Animal Experimentation Ethics Committee of the South–Central University of Nationalities (No. 2020-scuec-043) and were conducted according to the Animal Care and Use Committee guidelines of the South–Central University of Nationalities. Animals were housed in a room with controlled humidity (65 ± 5%) and temperature (25 ± 1 °C), under a 12/12-h light/dark cycle with free access to food and water for at least 1 week before the experiments. After the model was successfully established, **PM-Red-GSH** (100 μL, 200 μM) was injected through the tail vein, and the mice were anesthetized with isoflurane before fluorescence imaging using a Bruker *in vivo* imaging system. Whereafter, the mice were anesthetized and dissected to remove the mouse brain tissue, and a 300 μm section was prepared with a microtome.

2. Synthesis of Probe PM-Red-GSH

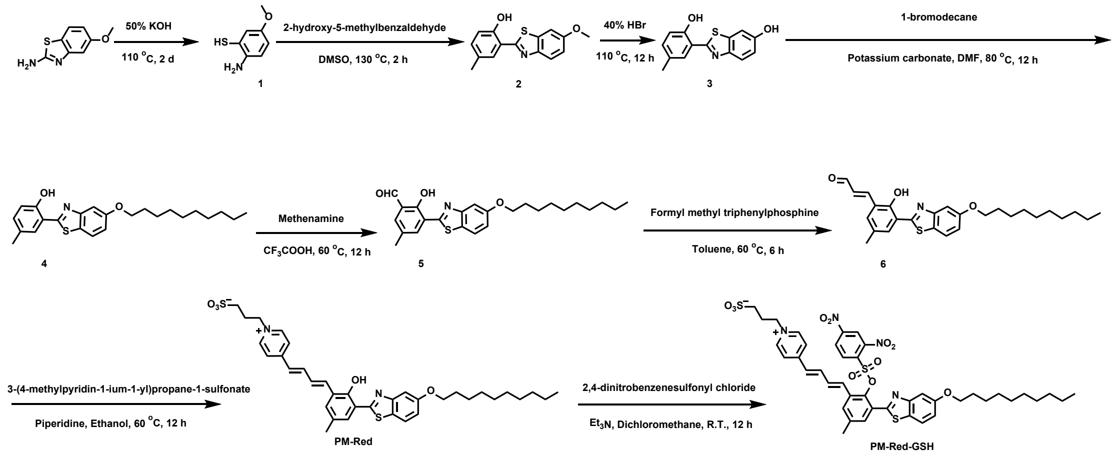

Scheme 1. The synthetic route for probe **PM-Red-GSH.**

Compound 1 was synthesized following the procedure described in previously published literature [1].

Synthesis of compound 2: 5-methylsalicylaldehyde (0.54 g, 4.0 mmol) and compound 1 (0.74 g, 4.8 mmol) were dissolved in 5 mL DMSO and place in a round bottom flask for reaction. Through TLC monitor the reaction until complete. After the reaction was completed, wash with water three times and extract with ethyl acetate three times, collect the organic phase and spin dry the solvent under reduced pressure, then purified by column chromatography (EA:PE = 1:8, Rf = 0.15) and obtained compound 2 (0.71, the yield was 65.99%). ^{1}H NMR (500 MHz, DMSO-d_6) δ 7.82 (d, *J* = 8.5 Hz, 1H), 7.40–7.36 (m, 2H), 7.10–7.06 (m, 2H), 6.85 (d, *J* = 8.3 Hz, 1H), 3.82 (s, 3H), 2.40 (s, 3H). ^{13}C{^{1}H} NMR (125 MHz, DMSO-d_6) δ 162.55, 156.59, 153.40, 147.41, 135.59, 132.24, 130.73, 128.88, 121.75, 118.64, 115.71, 113.99, 106.59, 54.84, 21.21.

Synthesis of compound 3: Compound 2 was dissolved in 40 mL 40% HBr solution, Stir the reaction overnight at 110 °C. Through TLC monitor the reaction until complete, then adjust the pH = 6 using a 1 mol/L NaOH solution, subsequently, the aqueous phase was adjusted to pH = 7 using a saturated $NaHCO_3$ solution. A light yellow solid was precipitated and filtered under reduced pressure, collected and recrystallized from ether to obtain white solid compound 3 (0.36 g, the yield was 69.23%).^{1}H NMR (500 MHz, DMSO – d_6) δ 8.64 (s, 1H), 7.79 (d, J = 8.7 Hz, 1H), 7.45 (d, J = 1.9 Hz, 1H), 7.39–7.36 (m, 1H), 7.10–7.05 (m, 1H), 6.91–6.84 (m, 2H), 2.42–2.38 (m, 3H). ^{13}C{^{1}H} NMR (125 MHz, DMSO-d_6) δ 162.46, 154.53, 153.40, 146.96, 135.73, 132.24, 130.73, 128.88, 122.54, 118.64, 116.28, 115.71, 108.92, 21.21.

Synthesis of compound 4: Compound 3 (0.10 g, 0.4 mmol) and K_2CO_3 (0.17 g, 1.2 mmol) and 10 mL DMF were placed in a round bottom flask, raise the temperature to 80 °C, then add 0.8 mmol of bromodecane to continue the reaction and stir overnight. After the reaction, the mixtures were quenched by water, extract with ethyl acetate three times, evaporated, and separated using silica gel chromatography (EA:PA = 1:3, Rf = 0.12) to acquire compound 4 as white powder (0.11 g, the yield was 73.33%). ^{1}H NMR (500 MHz, DMSO-d_6) δ 7.85 (d, *J* = 8.3 Hz, 1H), 7.37 (d, *J* = 1.8 Hz, 1H), 7.33 (d, *J* = 2.1 Hz, 1H), 7.10 – 7.06 (m, 1H), 7.02 (dd, *J* = 8.3, 1.9 Hz, 1H), 6.85 (d, *J* = 8.3 Hz, 1H), 4.01 (t, *J* = 6.3 Hz, 2H), 2.40 (s, 3H), 1.75 (tt, *J* = 7.5, 6.3 Hz, 2H), 1.44 (dq, *J* = 8.0, 7.0 Hz, 2H), 1.32 – 1.25 (m, 12H), 0.92 – 0.86 (m, 3H). ^{13}C{^{1}H} NMR (125 MHz, DMSO-d_6) δ 162.90, 158.20, 153.26, 150.83, 132.24, 132.20, 130.73, 128.88, 123.52, 118.64, 115.71, 115.59, 102.94, 68.54, 31.77, 29.59, 29.38, 29.28, 29.18, 29.14, 26.31, 22.65, 21.21, 14.05.

Synthesis of compound 5: Compound 4 (0.71 g,1.8 mmol), hexamethylenetetramine (0.55 g, 3.9 mmol) were dissolved in 15 mL trifluoroacetic acid. The mixture was stirred and refluxed at 80 °C for 12 h. After the reaction is complete, adjust the pH value with potassium hydroxide, until the solid is completely precipitated, filter and wash with water, evaporated, and separated using silica gel chromatography (EA:PA = 1:5, Rf = 0.11) to acquire compound 5 as yellow powder (0.52 g, the yield was 68.42%).^{1}H NMR (500 MHz, DMSO-d_6) δ 7.86 (d, *J* = 8.2 Hz, 1H), 7.57 (d, *J* = 2.5 Hz, 1H), 7.48 (d, *J* = 2.1 Hz, 1H), 7.33 (d, *J* = 2.2 Hz, 1H), 7.02 (dd, *J* = 8.3, 1.9 Hz, 1H), 4.01 (t, *J* = 6.3 Hz, 2H), 2.39 (s, 3H), 1.75 (tt, *J* = 7.5, 6.3 Hz, 2H), 1.44 (dq, *J* = 8.0, 7.0 Hz, 2H), 1.33–1.25 (m, 12H), 0.92–0.86 (m, 3H). ^{13}C{^{1}H} NMR (125 MHz, DMSO-d_6) δ 163.58, 158.20, 157.70, 150.73, 132.83, 132.22, 132.20, 131.90, 123.52, 122.48, 119.12, 115.59, 102.94, 68.54, 31.77, 29.59, 29.38, 29.28, 29.18, 2914, 26.31, 22.65, 21.14, 14.05.

Synthesis of compound 6: Compound 5 was dissolved in 20 mL methylbenzene, then add (formylmethylene)triphenylphosphine (1.86 g, 6.1 mmol) and stir at 60 °C for 6 h. After the reaction is complete, removing toluene by vacuum distillation and separated using silica gel chromatography (EA:PA = 1:5, Rf = 0.08) to acquire compound 6 as yellow powder (1.32 g, the yield was 48.71%).^{1}H NMR (500 MHz, DMSO-d_6) δ 9.66 (dd, *J* = 7.0, 1.3 Hz, 1H), 7.86 (d, *J* = 8.2 Hz, 1H), 7.52 (dd, *J* = 16.1, 1.3 Hz, 1H), 7.37–7.31 (m, 3H), 7.02 (dd, *J* = 8.3, 1.9 Hz, 1H), 6.72–6.67 (m, 1H), 4.01 (t, *J* = 6.3 Hz, 2H), 2.39 (s, 3H), 1.75 (tt, *J* = 7.5, 6.3 Hz, 2H), 1.44 (dq, *J* = 8.0, 7.0 Hz, 2H), 1.32–1.26 (m, 12H), 0.92 – 0.86 (m, 3H). ^{13}C{^{1}H} NMR (125 MHz, DMSO-d_6) δ 162.42, 158.20, 153.73, 150.73, 149.61, 132.22, 132.17, 130.85, 129.91, 124.87, 124.28, 123.52, 117.79, 115.59, 102.94, 68.54, 31.77, 29.59, 29.38, 29.28, 29.18, 29.14, 26.31, 22.65, 21.13, 14.05.

Synthesis of PM-RED : Compound 6 (0.45 g, 1 mmol) and 1-(3-sulfopropyl) pyridinium hydroxide (0.2 g, 1 mmol) were dissolved in 15 mL ethanol, then added 2 drops of piperidine and stir the reaction at 60 °C, and monitor the reaction through TLC until complete. Upon completion of the reaction, the mixture was allowed to cool to room temperature, followed by solvent removal under reduced pressure, and the resulting product was subsequently purified by column chromatography (DCM:MeOH = 30:1, Rf = 0.16). Finally, 0.22 g of fluorophore **PM-RED** was obtained with a yield of 33.85%.^{1}H NMR (500 MHz, DMSO – d_6) δ 8.99–8.95 (m, 2H), 8.19–8.14 (m, 2H), 7.86 (d, *J* = 8.2 Hz, 1H), 7.46–7.20 (m, 4H), 7.04–6.95 (m, 2H), 6.85–6.74 (m, 2H), 4.74 (t, *J* = 7.1 Hz, 2H), 4.01 (t, *J* = 6.3 Hz, 2H), 2.42–2.20 (m, 5H), 1.75 (tt, *J* = 7.5, 6.3 Hz, 2H), 1.51–1.40 (m, 4H), 1.34–1.24 (m, 13H), 0.92–0.86 (m, 3H). ^{13}C{^{1}H} NMR (125 MHz, DMSO-d_6) δ 162.07, 157.61, 152.11, 150.55, 146.48, 145.13, 135.33, 133.37, 132.43, 131.77, 130.93, 130.45, 130.30, 130.01, 125.53, 124.10, 124.04, 117.28, 115.58, 102.05, 68.48, 59.60, 57.99, 31.74, 29.33, 29.29, 29.27, 29.25, 29.17, 26.25, 24.64, 22.67, 21.13, 14.06.

Synthesis of probe PM-Red-GSH: Under the nitrogen atmosphere, a mixture of PM-Red (648 mg, 1 mmol), 2,4-Dinitrobenzenesulfonyl chloride (319 mg, 1.2 mmol), and Triethylamine (101 mg, 1 mmol) in 15 mL CH_2Cl_2 was stirred at room-temperature for 12 h. After completion, the reaction mixture was cooled to room temperature and purified via column chromatography (DCM:MeOH = 30:1, Rf = 0.08). Finally, obtained 252.6 mg pure of probe **PM-Red-GSH**, the yield 33.85%.^{1}H NMR (500 MHz, DMSO-d_6) δ 9.00- 8.95 (m, 2H), 8.89 (d, *J* = 1.9 Hz, 1H), 8.53 (dd, *J* = 8.1, 1.9 Hz, 1H), 8.33 (d, *J* = 8.0 Hz, 1H), 8.19–8.13 (m, 2H), 7.84 (d, *J* = 8.3 Hz, 1H), 7.57–7.53 (m, 2H), 7.40 (d, *J* = 2.0 Hz, 2H), 7.16–7.06 (m, 2H), 6.89–6.77 (m, 2H), 4.66 (t, *J* = 7.2 Hz, 2H), 4.01 (t, *J* = 6.3 Hz, 2H), 2.37–2.22 (m, 5H), 1.75 (tt, *J* = 7.4, 6.3 Hz, 2H), 1.52–1.39 (m, 4H), 1.36–1.27 (m, 12H), 1.27–1.21 (m, 3H), 0.94–0.85 (m, 3H). ^{13}C{^{1}H} NMR (125 MHz, DMSO-d_6) δ 162.15, 160.74, 157.66, 151.01, 148.50, 147.57, 146.78, 146.11, 145.21, 136.47, 135.52, 132.48, 132.36, 132.20, 131.70, 131.57, 131.48, 130.21, 130.18, 128.56, 127.62, 124.47, 124.14, 121.36, 120.95, 115.96, 102.21, 68.76, 60.44,

59.40, 31.82, 29.57, 29.54, 29.40, 29.37, 26.41, 24.47, 22.71, 21.15, 14.09. HRMS cacld for [M + H+]: 879.2398. Found: 879.2406.

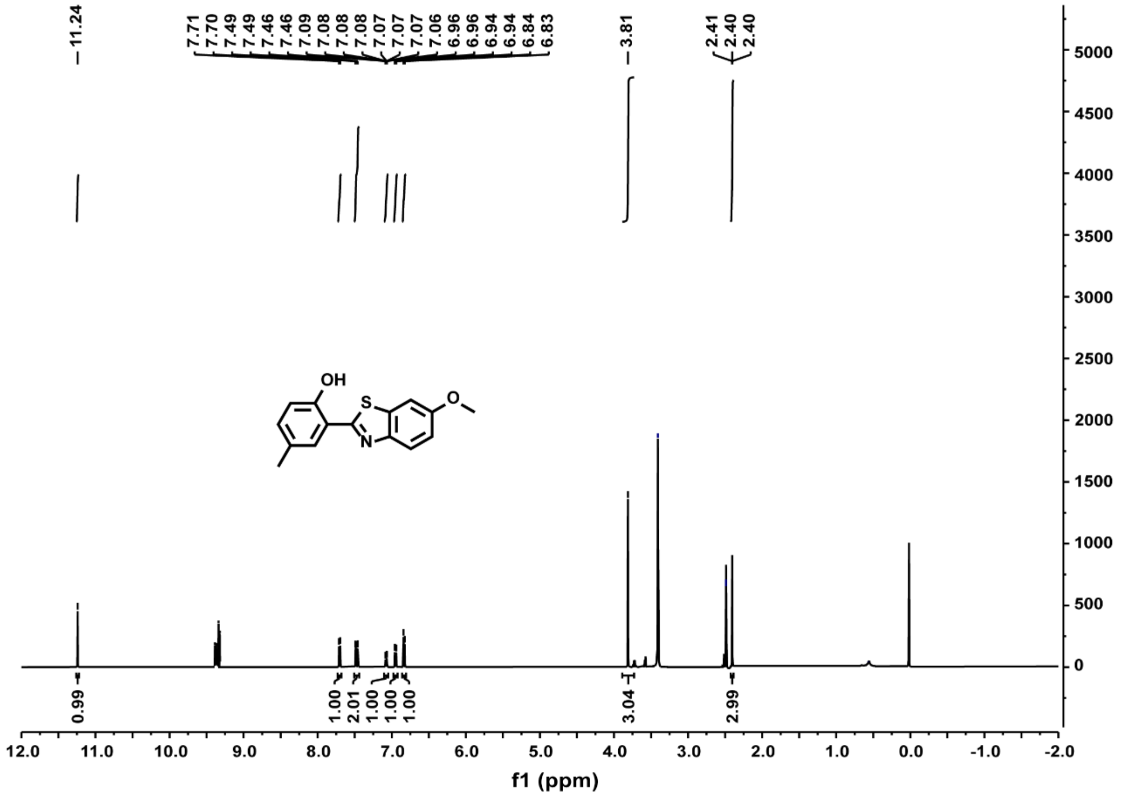

Figure S1. ^{1} HNMR spectrum of compound 2 in DMSO-*d*6 (500 MHz).

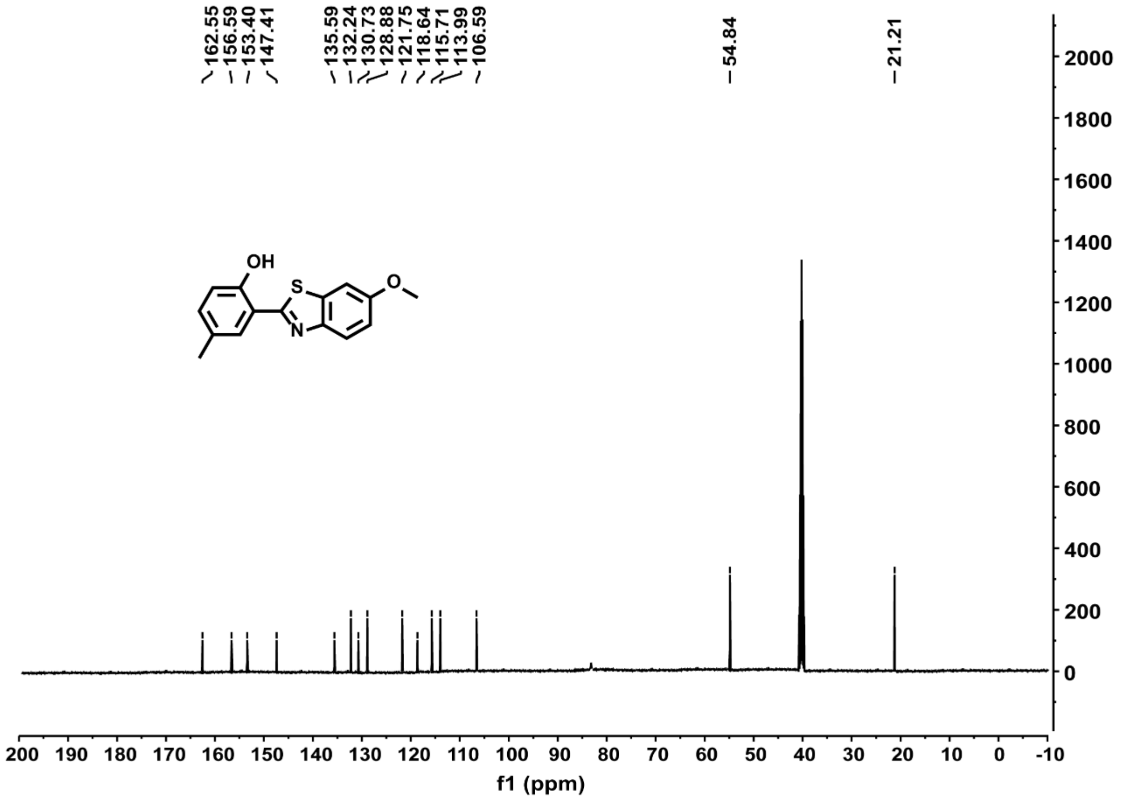

Figure S2. ^{13}C{^{1}H} NMR spectrum of compound 2 in DMSO-*d*6 (125 MHz).

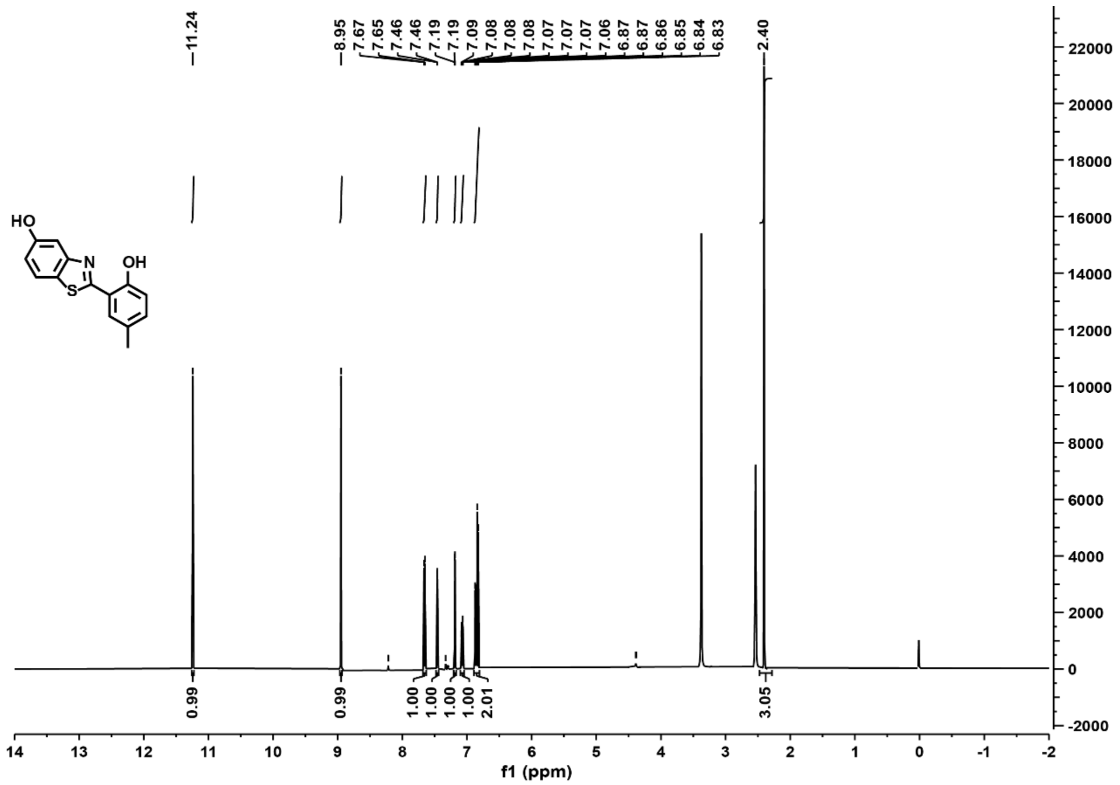

Figure S3. [1] HNMR spectrum of compound 3 in DMSO-d_6 (500 MHz).

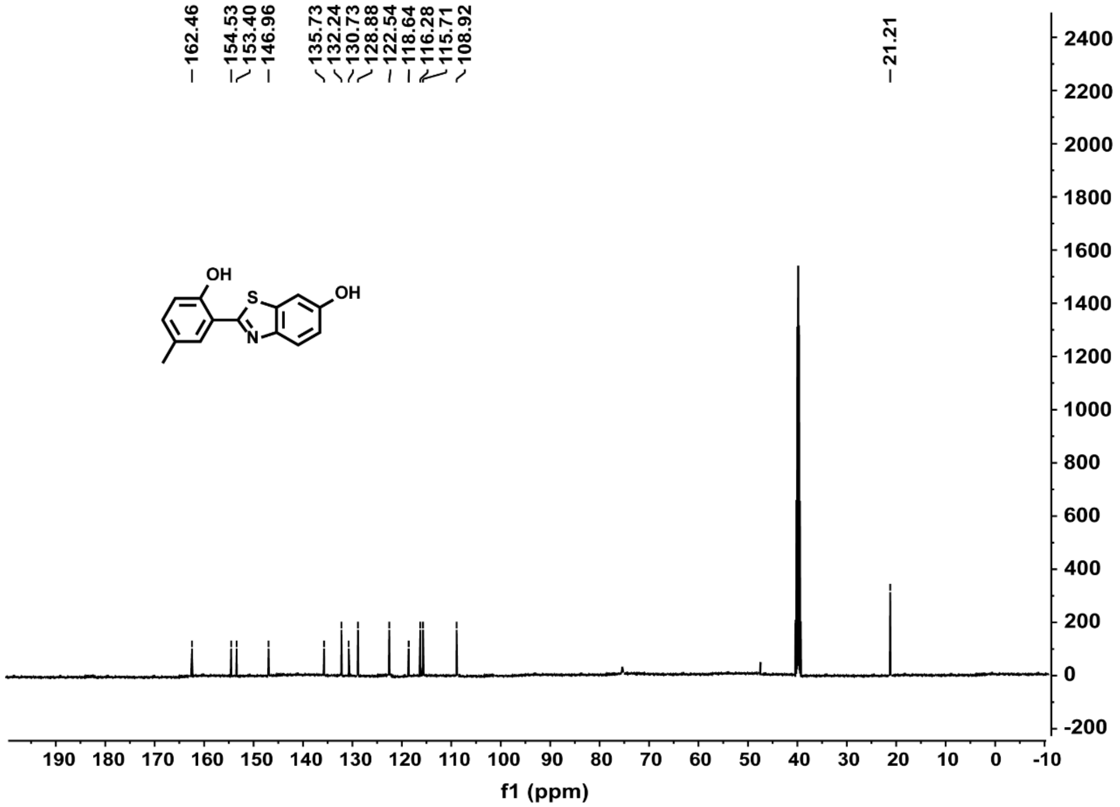

Figure S4. $^{13}C\{^1H\}$ NMR spectrum of compound 3 in DMSO-d_6 (125 MHz).

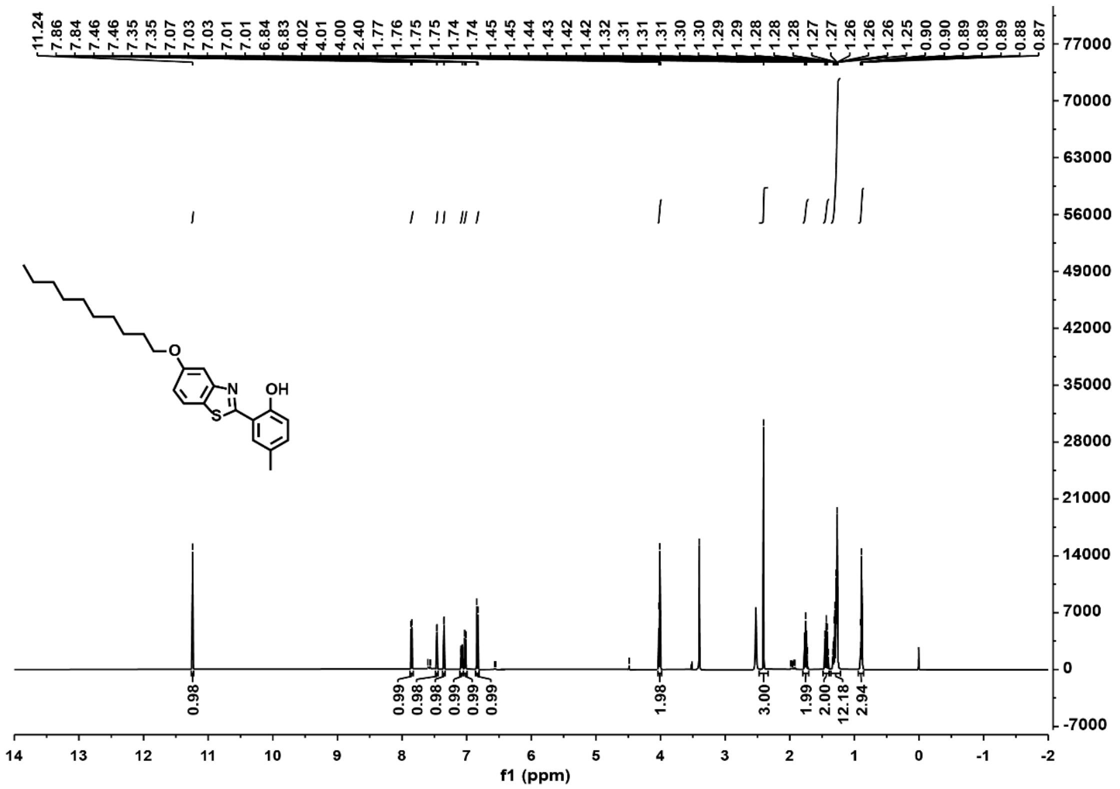

Figure S5. ^{1}HNMR spectrum of compound 4 in DMSO-d_6 (500 MHz).

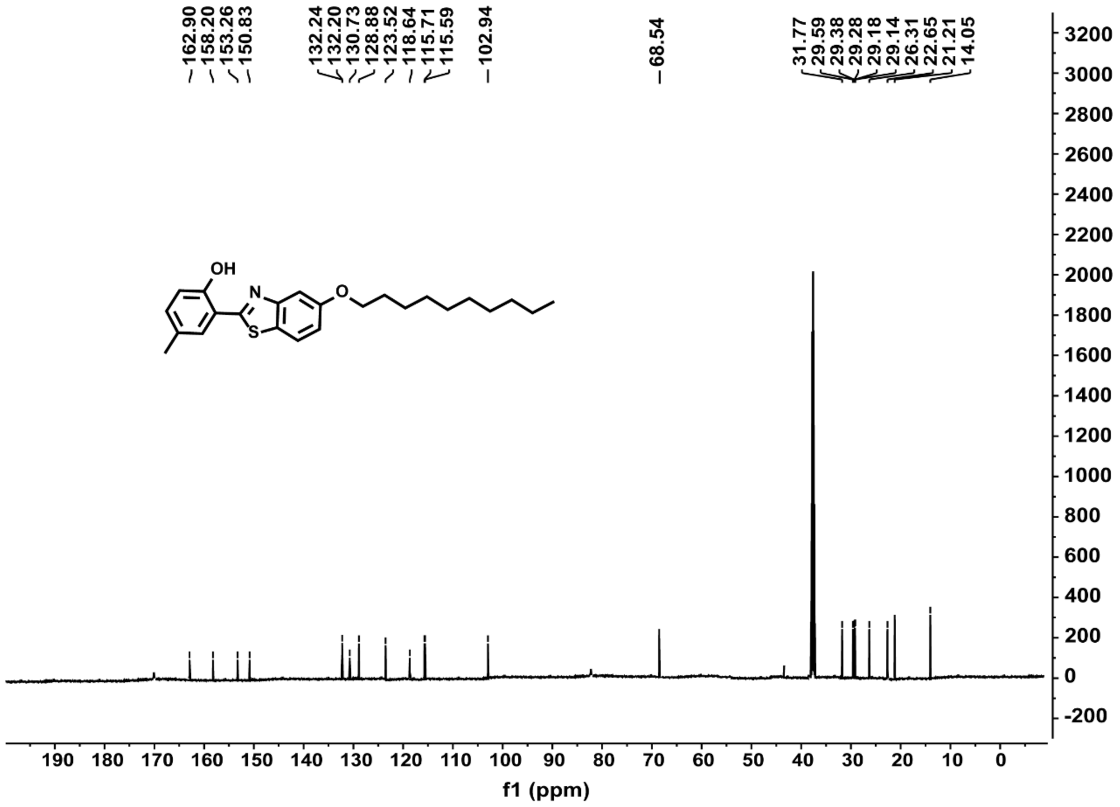

Figure S6. ^{13}C{^{1}H} NMR spectrum of compound 4 in DMSO-d_6 (125 MHz).

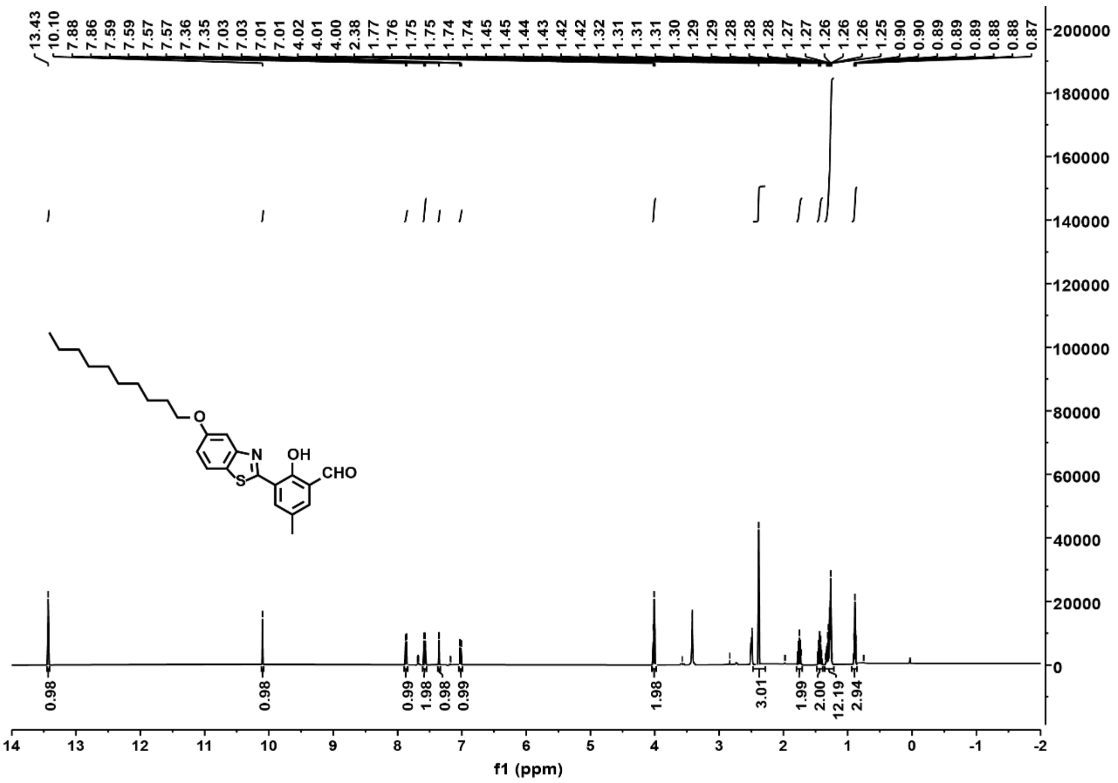

Figure S7. ^{1} HNMR spectrum of compound 5 in DMSO-*d*$_6$ (500 MHz).

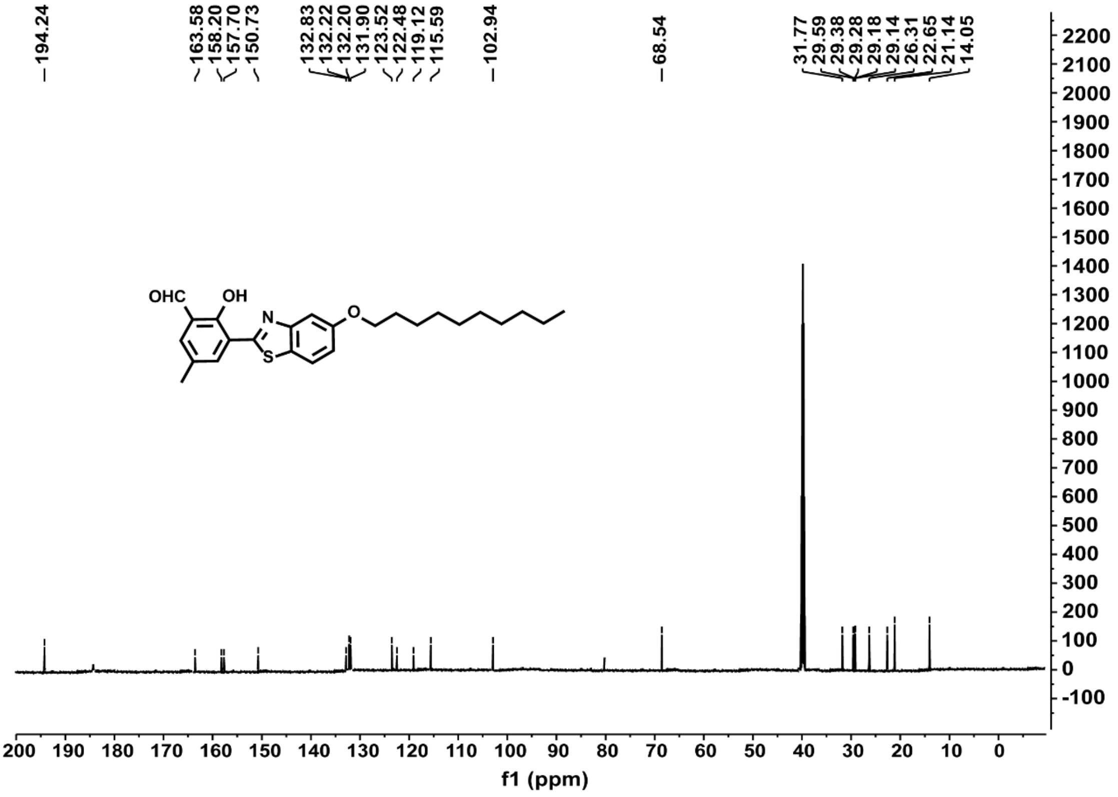

Figure S8. $^{13}C\{^1H\}$ NMR spectrum of compound 5 in DMSO-*d*$_6$ (125 MHz).

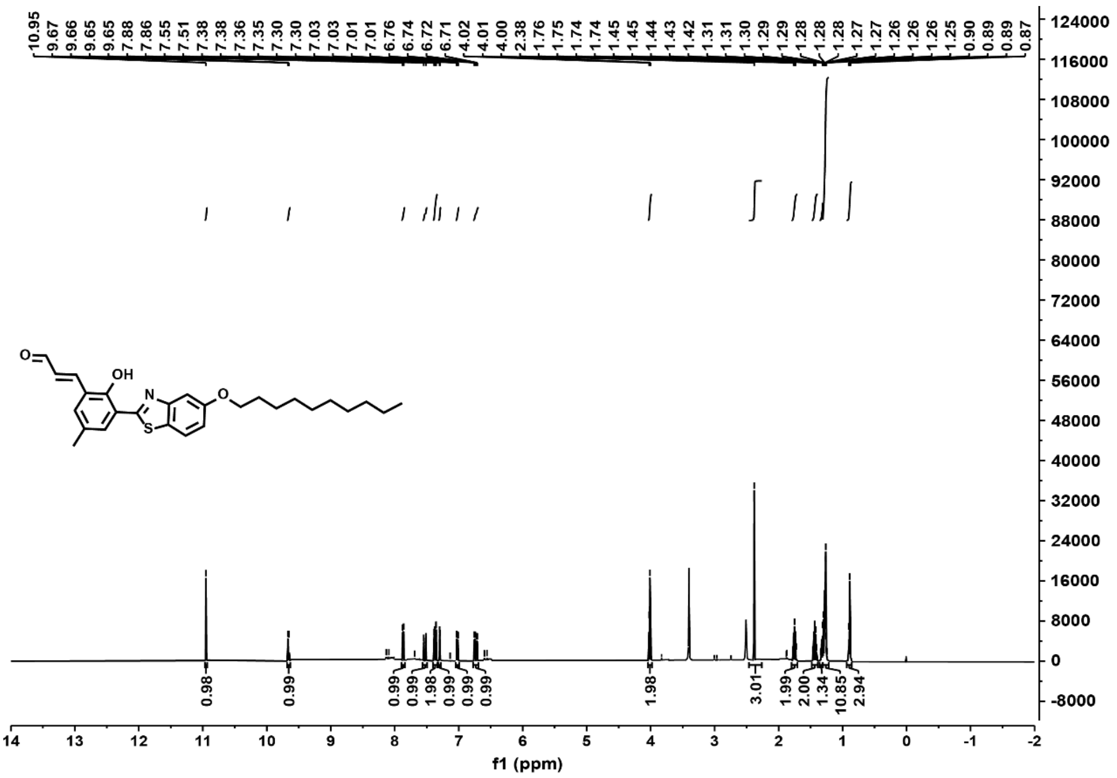

Figure S9. [1] HNMR spectrum of compound 6 in DMSO-d_6 (500 MHz).

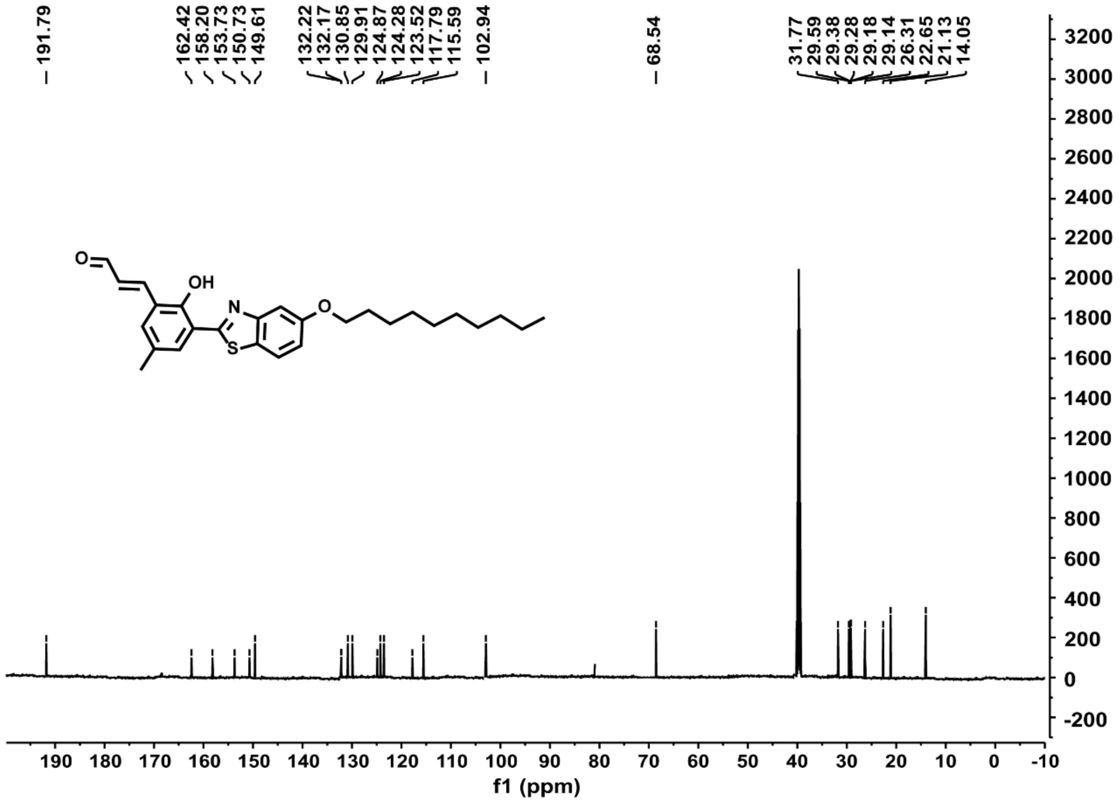

Figure S10. $^{13}C\{^1H\}$ NMR spectrum of compound 6 in DMSO-d_6 (125 MHz).

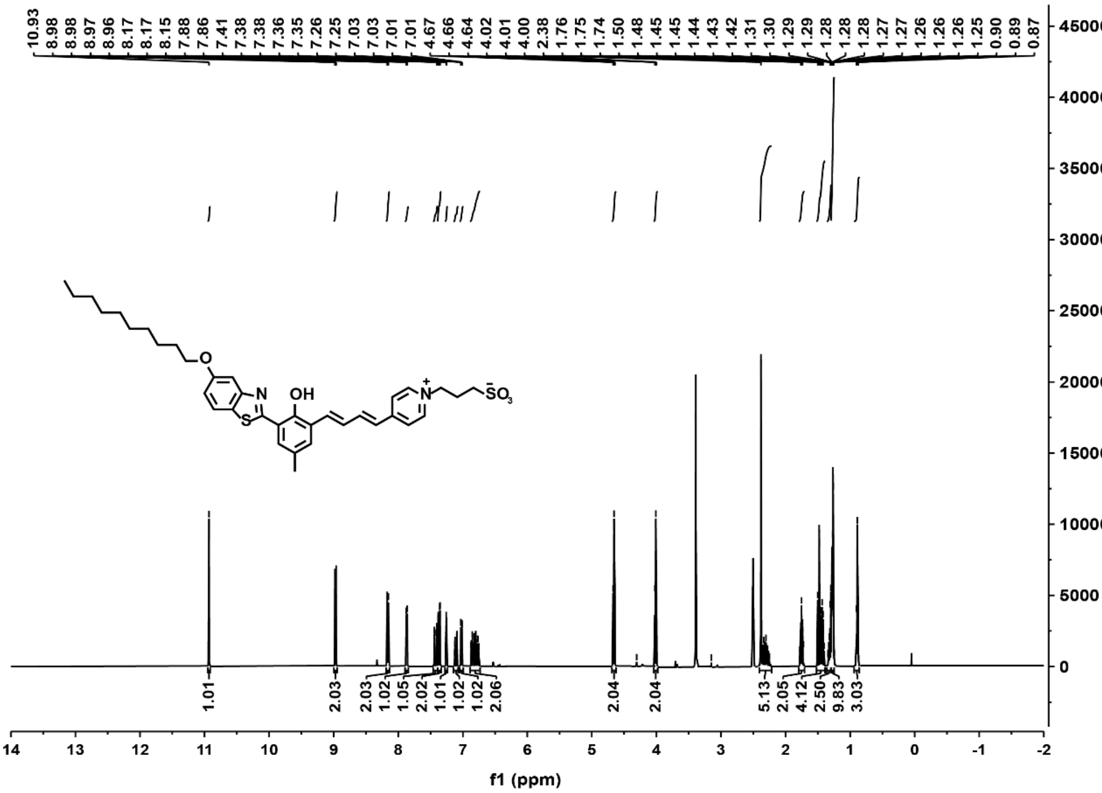

Figure S11. ^{1} HNMR spectrum of compound PM-Red in DMSO-*d₆* (500 MHz).

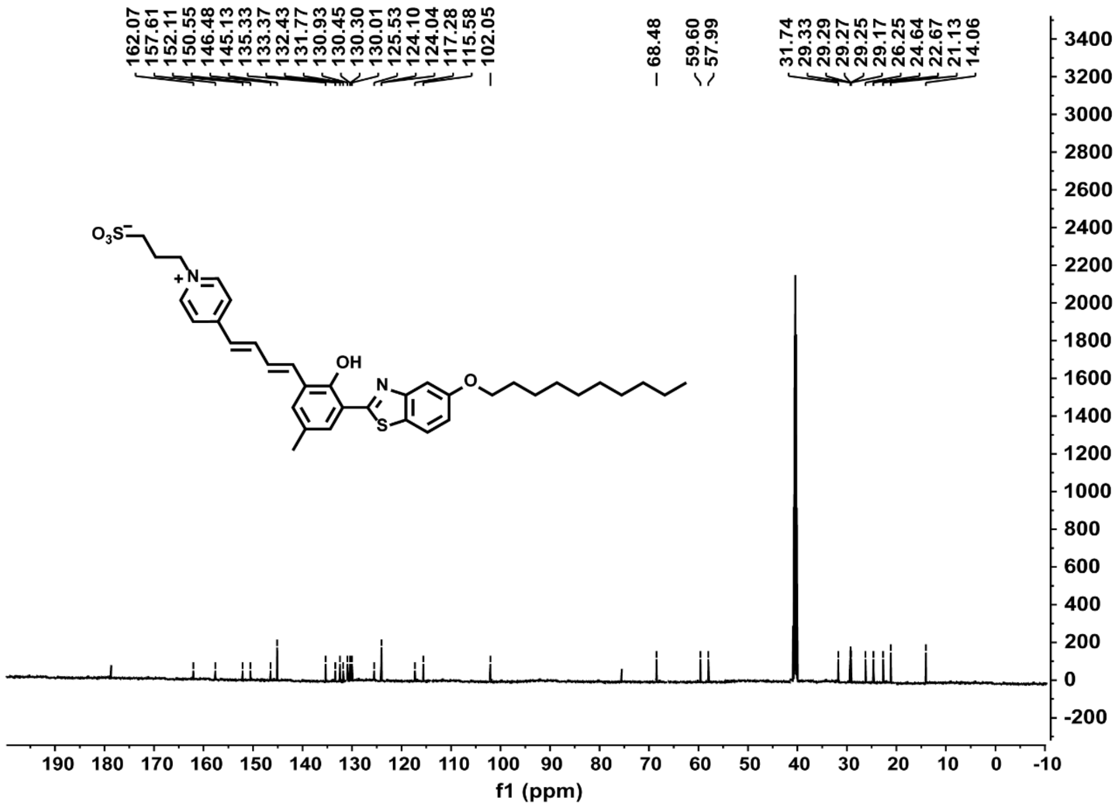

Figure S12. $^{13}C\{^1H\}$ NMR spectrum of compound PM-Red in DMSO-*d₆* (125 MHz).

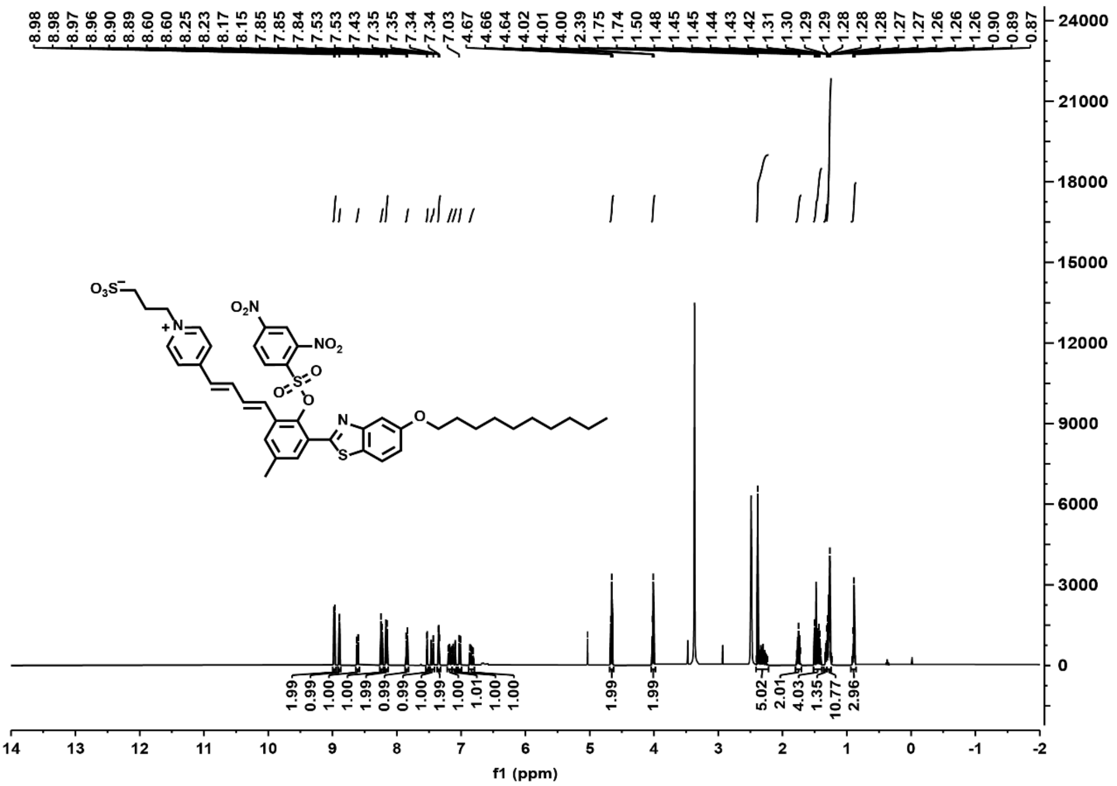

Figure S13. [1] HNMR spectrum of compound PM-Red-GSH in DMSO-*d*6 (500 MHz).

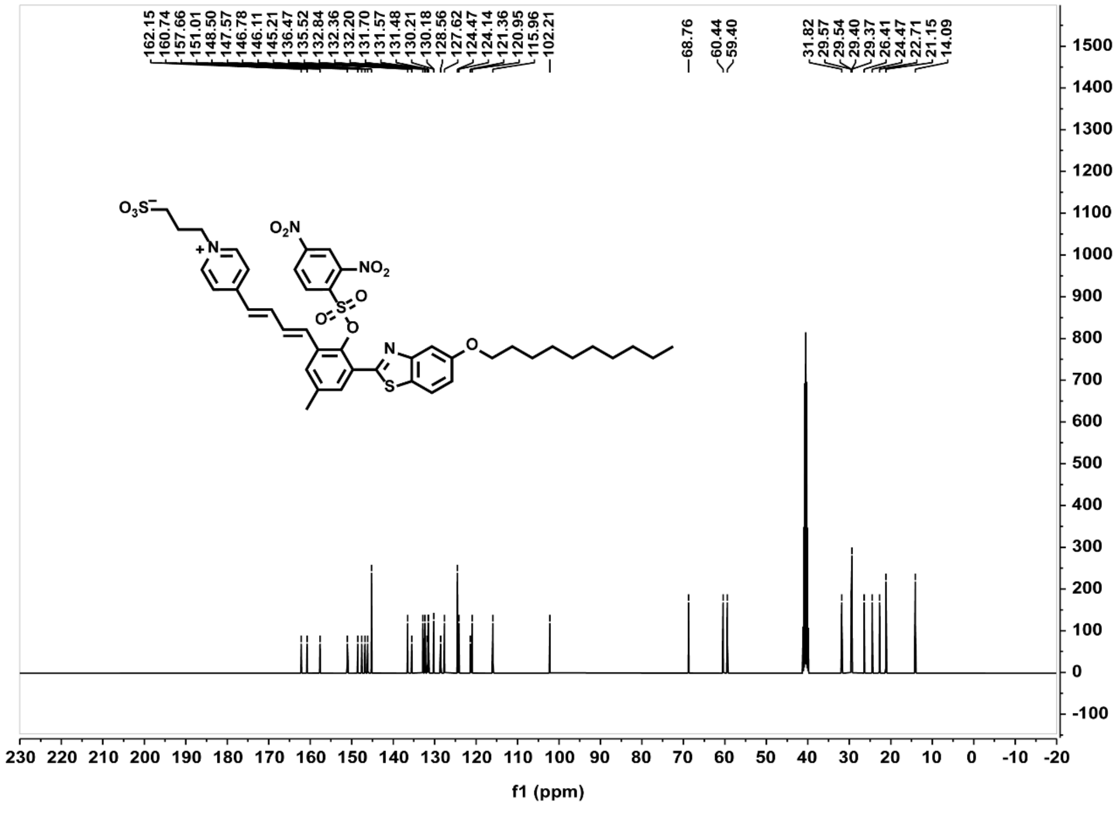

Figure S14. ^{13}C{^{1}H} NMR spectrum of compound PM-Red-GSH in DMSO-*d*6 (125 MHz).

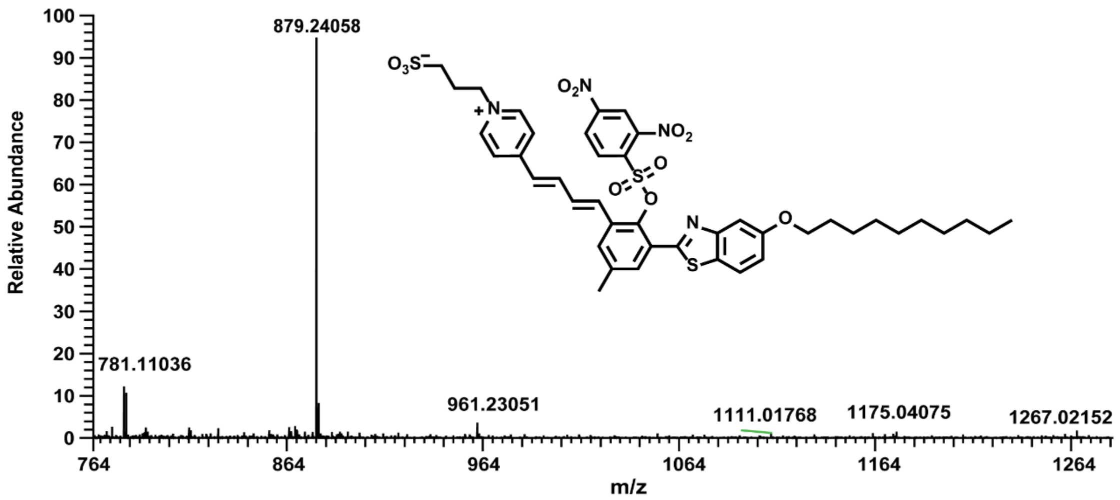

Figure S15. High-resolution mass spectrum of PM-Red-GSH.

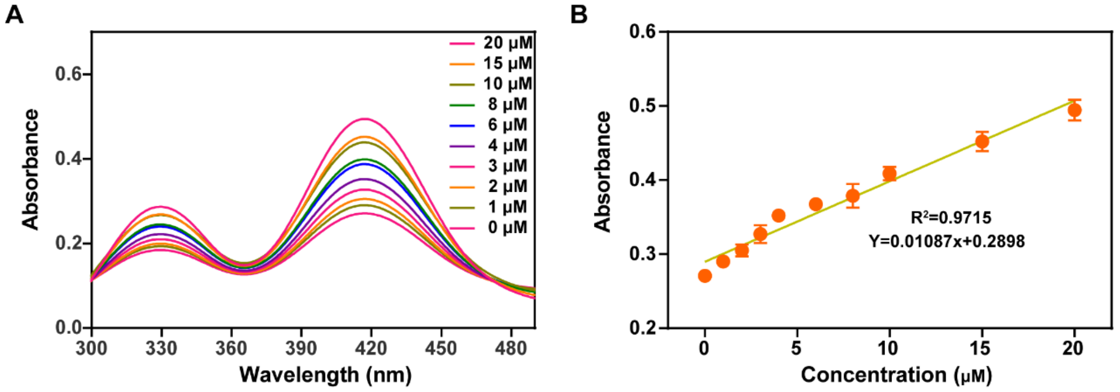

Figure S16. (**A**) The UV -*vis* absorption of PM-Red-GSH（1, 2, 3, 4, 6, 8, 10, 15, 20 μM). (**B**) Liner relationship of the absorbance at 420 nm with the concentrations of PM-Red-GSH.

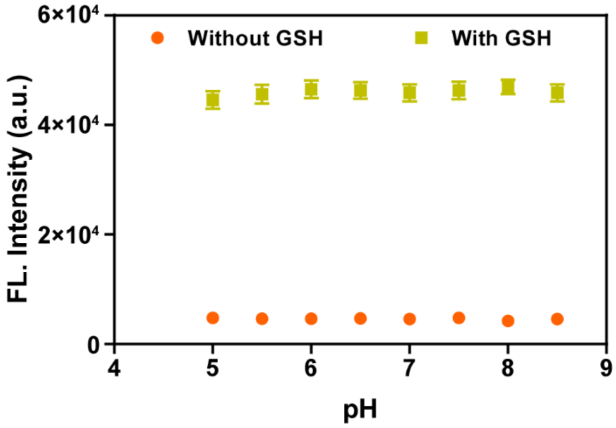

Figure S17. pH effects of the probe and the reaction between PM-Red-GSH and GSH. Fluorescence changes of PM-Red-GSH with or without GSH in solution with different pH values (5.0–8.5). λ_{ex} = 420 nm, λ_{em} = 715 nm.

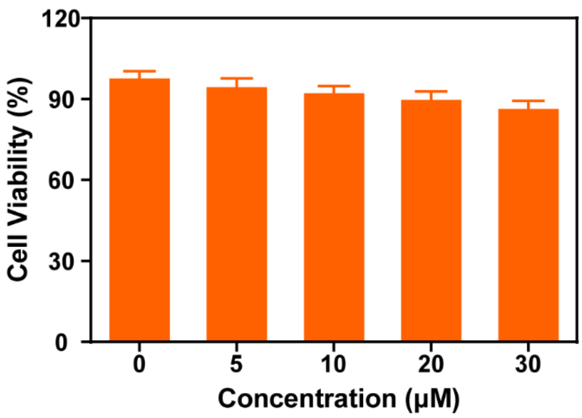

Figure S18. Cytotoxicity of **PM-Red-GSH**. BV-2 cells were incubated with various amount (0–30 μM) of **PM-Red-GSH** for 24 h and measured by MTT method.

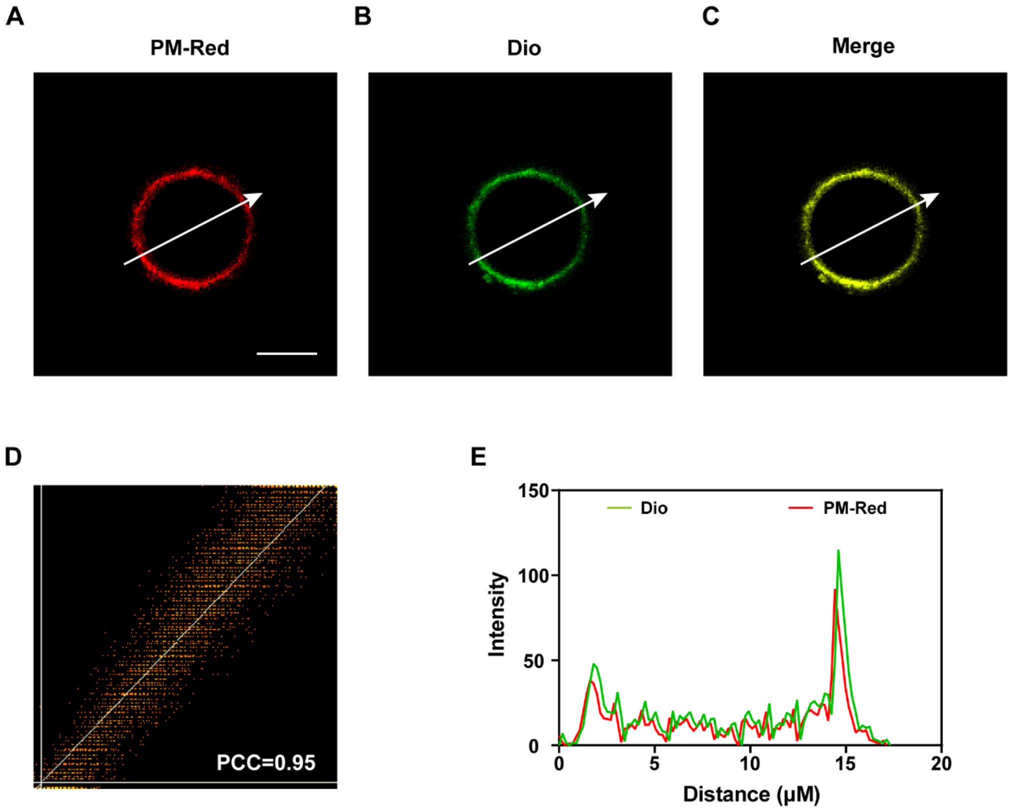

Figure S19. Confocal images of BV-2 cells after co-staining with 10 μM PM-Red and 10 μM Dio, The red channel image was collected at 625–725 nm (λ_{ex} = 420 nm) for PM-Red. The green channel image was collected at 500–550 nm (λ_{ex} = 488 nm) for Dio. Scale bar = 20 μm.

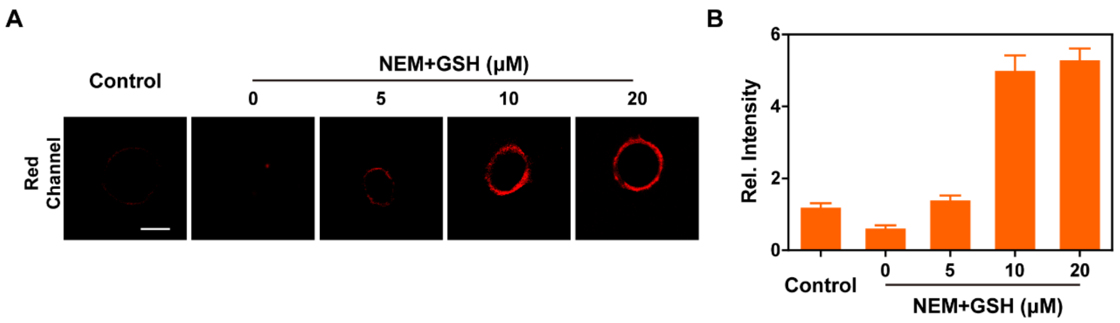

Figure S20. (**A**) Confocal images of BV-2 cells incubated with probe PM-Red-GSH (10 μM) in control group (only probe), PM-Red-GSH (10 μM) for 10 min and then, NEM (0.5 mM), GSH (0, 5, 10, 20 μM) for 40 min. (**B**) Histograms of average fluorescence intensity of (**A**). Scale bar = 20 μm.

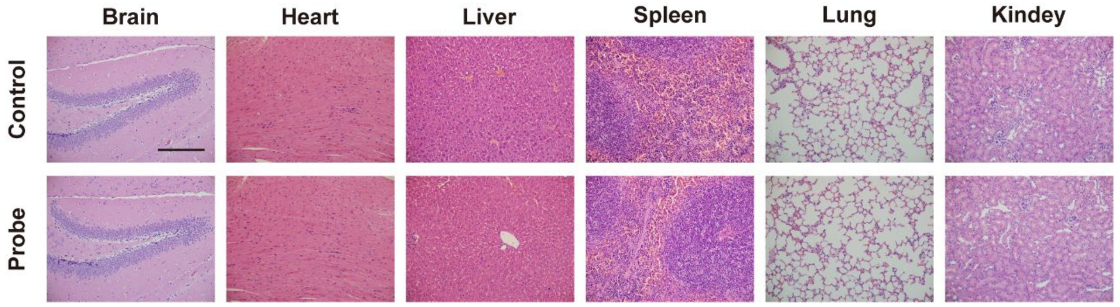

Figure S21. H&E-stained sections of major organs s collected from the control group and probe PM-Red-GSH (100 μL, 200 μM) treated group. Scale bar: 50 mm.

Table S1. Photophysical properties of PM-RED-GSH under different conditions.

Dye	λ_{max}^{abs} (nm) [a]	ε ($M^{-1}cm^{-1}$) [b]	λ_{abs}^{em} (nm) [c]	Φ [d]
**PM-RED-GSH**	420	5.5×10^4	-	<0.001
**PM-RED-GSH+GSH**	420	3.5×10^4	715	0.114
**PM-RED**	420	3.9×10^4	715	0.211

[a] maximum absorption wavelength. [b] molar absorptivity. [c] maximum fluorescence emission wavelength. [d] quantum yield.

Table S2. Comparison of the present probe with other reported GSH fluorescence probe.

Structure of Probe	λ_{abs}/nm	λ_{em}/nm	Stokes Shift/nm	Limit of Detection	Response Time	Buffer Solution	Molar Absorption Coefficient (10^4 M^{-1} cm^{-1})	Quantum Yield	Ref.
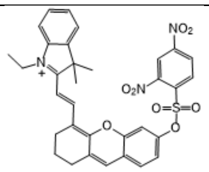	690	716	26	/	Cys: 60 s	PBS/CH_3CN = 7:3	7.9 [a]	0.36 [a]	[2]
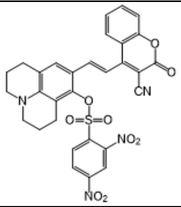	601	679	78	Cys: 7.9 nM Hcy: 10.2 nM GSH: 4.2 nM	Cys: 12 min Hcy: 15 min GSH: 9 min	DMSO/PBS = 4:1	/	/	[3]

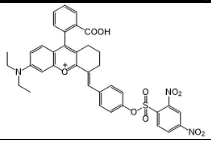	550	660	110	Cys: 43 μM	Cys: 10 min	PB/DMSO = 8:2	/	0.0216 [a]	[4]
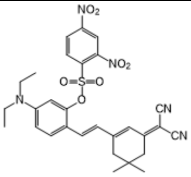	560	680	120	Cys: 36.93 nM<br>Hcy: 65.03 nM<br>GSH: 32.56 nM	Cys: 6 min<br>Hcy: 14 min<br>GSH: 12 min	PBS/ethanol = 1:1	/	0.2971 [a]	[5]
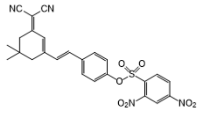	550	676	126	Cys: 12.1 nM<br>Hcy: 14.5 nM<br>GSH: 11.9 nM	Cys/Hcy/GSH: 6 min	PBS/DMSO = 1:1	4.2 [a]	/	[6]
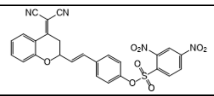	560	690	130	GSH: 18 nM	GSH: 5 min	PBS/DMSO = 1:1	/	/	[7]
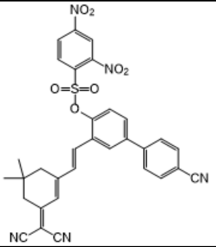	536	670	134	Cys: 36.0 nM<br>Hcy: 39.0 nM<br>GSH: 48.0 nM	Cys: 6 min<br>Hcy: 4.5 min<br>GSH: 6 min	PBS with 1.0 mM CTAB	/	/	[8]
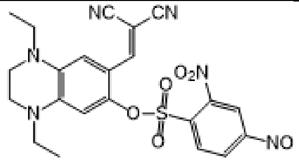	472	619	147	Cys: 15 nM<br>Hcy: 50 nM<br>GSH: 20 nM	Cys/Hcy/GSH: 100 min	HEPES/CH_3CN = 7:3	/	/	[9]
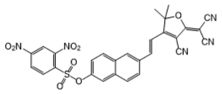	480	640	160	Cys: 0.11 μM<br>Hcy: 0.47 μM<br>GSH: 0.35 μM	Cys/Hcy/GSH: 180 min	HEPES/ethanol = 1:1	4.87 [a]	0.0379[a]	[10]
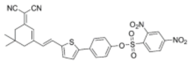	470	668	198	Cys: 28 nM<br>Hcy: 22 nM<br>GSH: 24 nM	Cys: 25 min<br>Hcy: 30 min<br>GSH: 45 min	PB/DMSO = 7:3	4.71 [a]<br>3.96 [b]	0.37 [a]<br>0.04 [b]	[11]
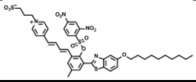	425	715	290		GSH:30 min	PBS/CH_3CN = 5:5		<0.001 [a]<br>0.114 [b]	This work

a: date for fluorophore, b: date for probe.

References

1. Jian, Z.; Ye, Y.; Yu, K.; Zhu, W.H.; Wang, J.; Xiong, X.; Li, C.; Gu, L. Ratiometric two-photon fluorescent probe for imaging hydrogen peroxide during stroke-induced ferroptosis. *Sens. Actuators B Chem.* **2024**, *417*, 136064.
2. Yuan, L.; Lin, W.Y.; Zhao, S.; Gao, W.S.; Chen, B.; He, L.W.; Zhu, S.S. A unique approach to development of near-infrared fluorescent sensors for in vivo imaging. *J. Am. Chem. Soc.* **2012**, *134*, 13510–13523.
3. Wang, J.P.; Zhang, F.; Yang, L.; Wang, B.H.; Song, X.Z. A red-emitting fluorescent probe for sensing and imaging biothiols in living cells. *J. Lumin.* **2021**, *234*, 117994.
4. Liu, K.Y.; Shang, H.M.; Kong, X.Q.; Lin, W.Y. A novel near-infrared fluorescent probe with large Stokes shift for biothiols and the application for in vitro and in vivo fluorescence imaging. *J. Mater. Chem. B* **2017**, *5*, 3836–3841.
5. . Qian, M.; Zhang, L.W.; Wang, J.Y. A NIR fluorescent sensor for biothiols based on a dicyanoisophorone derivative with a large Stokes shift and high quantum yield. New J. *Chem.* **2019**, *43*, 9614–9622.

6. Wang, K.; Leng, T.H.; Liu, Y.J.; Wang, C.Y.; Shi, P.; Shen, Y.J.; Zhu, W.H. A novel near-infrared fluorescent probe with a large stokes shift forthe detection and imaging of biothiols. *Sens. Actuators B Chem.* **2017**, *248*, 338–345.
7. Li, M.; Wu, X.M.; Wang, Y.; Li, Y.S.; Zhu, W.H.; James, T.D. A near-infrared colorimetric fluorescent chemodosimeter for the detection of glutathione in living cells. *Chem. Commun.* **2014**, *50*, 1751–1753.
8. Ma, C.H.; Yan, D.L.; Hou, P.; Liu, X.B.; Wang, H.; Xia, C.H.; Li, G.; Chen, S. Bioimaging and sensing thiols in vivo and in tumor tissues based on a near-infrared fluorescent probe with large Stokes shift. *Molecules* **2023**, *28*, 5702.
9. Qi, F.P.; Liu, X.J.; Yang, L.; Yang, L.; Chen, W.Q.; Song, X.Z. A red-emitting fluorescent probe for biothiols detection with a large Stokes shift. *Tetrahedron* **2016**, *72*, 6909–6913.
10. Chen, D.G.; Long, Z.; Sun, Y.M.; Luo, Z.J.; Lou, X.D. A red-emission probe for intracellular biothiols imaging with a large Stokes shift. *J. Photochem. Photobiol. A* **2019**, *368*, 90–96.
11. Wu, Z.J.; Zhao, T.T.; Jiang, X.Y.; Xia, X.F.; Zhang, D.; Wang, F.Y.; Ren, X.M.; Wang, Z.; Ren, J.; Wang, E.F. A near-infrared fluorescent probe with a large Stokes shift for the detection and imaging of biothiols in vitro and in vivo. *Anal. Bioanal. Chem.* **2024**, *416*, 6485–6495.

Disclaimer/Publisher's Note: The statements, opinions and data contained in all publications are solely those of the individual author(s) and contributor(s) and not of MDPI and/or the editor(s). MDPI and/or the editor(s) disclaim responsibility for any injury to people or property resulting from any ideas, methods, instructions or products referred to in the content.
